# Treatment of low-risk prostate cancer: a retrospective study with 477 patients comparing external beam radiotherapy and I-125 seeds brachytherapy in terms of biochemical control and late side effects

**DOI:** 10.1007/s00066-020-01657-1

**Published:** 2020-07-08

**Authors:** Matthias Moll, Christopher Paschen, Alexandru Zaharie, Florian Berndl, Gregor Goldner

**Affiliations:** 1 Department of Radiation Oncology, Medical University of Vienna, Vienna, Austria; 2 Department of Urology, Medical University of Vienna, Vienna, Austria

**Keywords:** Permanent interstitial, Localized prostate cancer, Patient’s acceptance, Survival, Follow-up

## Abstract

**Purpose:**

The goal of our study was comparison of external beam radiotherapy (EBRT) and I‑125 seeds brachytherapy in terms of biochemical control and development of late gastrointestinal and genitourinary side effects.

**Patients and methods:**

477 low-risk prostate cancer patients treated between 2000 and 2019 at our department using either I‑125 seeds brachytherapy or EBRT with a dose of 74 or 78 Gy were reviewed for our analysis. 213 patients were treated with EBRT and 264 with seeds.

**Results:**

Patients were followed up yearly with a median follow-up of 70 (3–192) months. The biochemical no evidence of disease (bNED) rates after 5 years were 95% for both EBRT and seeds, and after 10 years 87% for EBRT and 94% for seeds using the Phoenix criteria, although no significant difference was observed. Concerning gastrointestinal side effects, EBRT showed significantly higher rates of RTOG grade ≥2 toxicity compared to seeds, but at no point in follow-up more than 15% of all patients. On the other hand, genitourinary side effects were significantly more prevalent in patients treated with seeds, with 40% RTOG grade ≥2 toxicity 12 months after treatment. Nevertheless, both types of side effects decreased over time.

**Conclusion:**

Both EBRT and seeds provide excellent biochemical control with bNED rates after 10 years of about 90%. In terms of side effects, patients treated with seeds show higher grades of genitourinary side effects, while patients treated with EBRT show higher grades of gastrointestinal side effects.

## Introduction

Low-risk prostate cancer can be treated with surgery, irradiation, or active surveillance. All of these treatment modalities achieve similar oncological results in terms of prostate cancer-specific survival [1].

At our department, external beam radiotherapy (EBRT) and I‑125 seeds brachytherapy are performed on a regular basis. The goal of this study is to show on the one hand that both techniques have provided similar results in terms of biochemical recurrence over a period of nearly 20 years, while including almost all of the low-risk patients treated here. On the other hand, we want to display the level of side effects during treatment, as high oncological levels of success shift the focus more and more towards side effects caused by different treatment modalities.

## Materials and methods

The study protocol was approved by the ethical review board of our medical university according to local law regulations (EK no.: 1991/2019).

All patients included were treated at our Department of Radiation Oncology between 01/2000 and 12/2019. Patients had to meet the following inclusion criteria:

1. Low-risk prostate cancer, defined using the National Comprehensive Cancer Network (NCCN classification) [2]: initial prostate-specific antigen (PSA) ≤10 ng/dl and pT1a‑c or cT2a and Gleason score 6 or less.
2. Localized cancer with a clinical stage of cNx/0 and cMx/0.
3. Primary local treatment either via EBRT with a total dose of 74 or 78 Gy and 2 Gy per fraction or via seeds.

Patients were informed about both EBRT and seeds brachytherapy. The final choice was made by the patient. However, if the decision for brachytherapy was made, the inclusion criteria recommended by the ESTRO [3] had to be fulfilled.

Patients received transperineal implantation of I‑125 seeds as monotherapy. Before implantation of seeds, a pre-planning, such as recommended by Battermann et al. [4], was performed. The prescribed dose was 145 Gy for the surrounding isodose according to the TG43 protocol [5]. The activity of the seeds was 0.43–0.46 mCi. All seed applications were performed by the same radiation oncologist. Patients received spinal anesthesia.

Definition of the clinical target volume was performed using CT and MRI for planning. The total prescribed dose was 74 or 78 Gy with 2 Gy per fraction, administered with 3D conformal radiotherapy or volume-modulated arc therapy (VMAT), depending on the state of the art at the time of treatment. The dose was prescribed to 95% of the PTV according to ICRU report 62 [6]. Due to the long timeframe of our study, safety margins differed from 5 mm with gold markers or 7 to 10 mm without. All patients received a rectal balloon. The irradiation was performed in supine position via either conformal four-field box 3D or VMAT technique.

Biochemical recurrence was defined as PSA nadir +2 ng/ml using the Phoenix criteria [7]. Patients were followed up 3 months after treatment, 12 months after, and every 12 months from that point on. PSA levels were recorded for every follow-up. Late gastrointestinal und genitourinary side effects were routinely assessed and recorded by the physician during follow-up using RTOG grading [8].

Statistical analysis was performed using GraphPad Prism 8 (GraphPad Software, San Diego, USA). A *p*-value of <0.05 was considered statistically significant. The Kaplan–Meier method was used to estimate bNED rates. The resulting curves were compared using the log-rank test. Side effects were analyzed using the Mann–Whitney U test.

## Results

Our retrospective analysis included 477 primary low-risk prostate cancer patients. 213 patients received EBRT, 146 with a total dose of 74 Gy and 67 with a total dose of 78 Gy. 264 patients were treated with seeds. Patient characteristics are displayed in Table 1.

**Table 1:** Patient characteristics

	74 Gy		78 Gy		EBRT total		I‑125 seeds		Total	
*n* *=*	146	31%	67	14%	213	45%	264	55%	477	100%
*T stage*										
1a/b	20	14%	10	15%	30	14%	0	0%	30	6%
1c	102	70%	50	75%	152	71%	213	81%	365	77%
2a	24	16%	7	10%	31	15%	51	19%	82	17%
*iPSA in ng/ml*										
Min	0.3		1.5		0.3		0.55		0.3	
Max	10		10		10		9.99		10	
Mean	6.4		6.3		6.4		6.4		6.4	
*Gleason score*										
<6	24	16%	2	3%	26	12%	18	7%	44	9%
6	122	84%	65	97%	187	88%	246	93%	433	91%
*ADT*										
Yes	64	44%	13	19%	77	36%	33	13%	110	23%
Median duration in months	6		6		6		5.5		6	
*Age in years*										
Min	54		56		54		49		49	
Max	82		80		82		84		84	
Mean	70		70		70		68		69	
*Follow-up*										
Min	3		3		3		3		3	
Max	192		108		192		181		192	
Mean in months	78		55		71		68		70	
*Technique*										
3D conformal	146	100%	39	58%	185	87%	0	0%	185	39%
VMAT	0	0%	28	42%	28	13%	0	0%	28	6%
Seeds	0	0%	0	0%	0	0%	264	100%	264	55%

*Min* minimum, *Max* maximum, *iPSA* initial prostate-specific antigen, *VMAT* volumetric modulated arc therapy

There were only minor differences between the groups regarding T stage, PSA levels, Gleason score, and age. Follow-up was longer for patients treated with seeds and 74 Gy, as 78 Gy and the use of VMAT became standard of care later on. 17% of all patients had a follow-up of 120 months or more. Another difference was the increased prescription of androgen deprivation therapy (ADT) in patients treated with 74 Gy compared to the other groups.

We performed an internal comparison between the bNED of EBRT with 74 Gy and 78 Gy. This analysis showed biochemical control rates of 93% for patients treated with 74 Gy and 98% for patients treated with 78 Gy after 5 years and 88% and 81% after 10 years for 74 Gy and 9 years for 78 Gy. The statistical analysis showed no significant difference between the two groups. Therefore, we decided to merge both into one EBRT group.

**Fig. 1:**
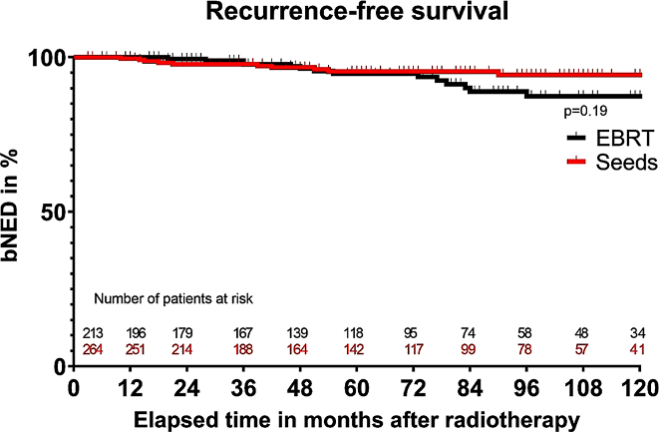
Recurrence-free survival after either external beam radiotherapy (EBRT) or seeds

The 5‑ and 10-year bNED rates for patients treated with EBRT were 94 and 87%, respectively. For patients treated with seeds, these rates were 95 and 94%, respectively (see Fig. 1). The latest reported biochemical recurrence occurred after 96 months of follow-up. 5 out of 10 biochemical failures after seed implantation were once again irradiated locally, whereas 1 out of 14 EBRT bNED failures was reirradiated locally.

Regarding survival, we were able to detect 36 deaths (15 after EBRT and 21 after BT). None of these were cancer related; therefore, cancer-specific survival was 100% for both treatments. Overall survival rates after 10 years were 88% for EBRT and 86% for BT, without a significant difference (*p* = 0.63).

Maximum late side effects at any point during treatment and follow-up are displayed in Table 2. It is notable that the only two patients reporting RTOG grade 4 genitourinary toxicity in the form of urinary retention that required surgical urological intervention were patients receiving brachytherapy. Overall, all subgroups tolerated the treatment well. On top of that, patients receiving EBRT reported in 61% of cases no gastrointestinal and in 43% no genitourinary side effects. For seeds, 68% of all patients reported no gastrointestinal side effects, but only 8.7% reported no genitourinary side effects. There was a significant difference between the 74 and 78 Gy groups concerning maximum genitourinary side effects (*p* = 0.01). No difference was found for maximum gastrointestinal side effects in EBRT. Comparing EBRT and seeds, we found significant differences regarding maximum late gastrointestinal and genitourinary side effects (*p* = 0.02 and *p* < 0.001, respectively).

**Table 2:** Maximum of late gastrointestinal (GI) and genitourinary (GU) side effects

Maximum of	Late GI side effects				Late GU side effects			
RTOG	74 Gy	78 Gy	EBRT	Seeds	74 Gy	78 Gy	EBRT	Seeds
Grade 0	63%	55%	61%	68%	48%	33%	43%	9%
Grade 1	13%	15%	14%	22%	28%	27%	28%	13%
Grade 2	22%	30%	25%	11%	19%	36%	24%	73%
Grade 3 or >3	1%	0%	1%	0%	5%	4%	5%	0%
*n* =	145	67	212	263	145	67	212	263

**Fig. 2:**
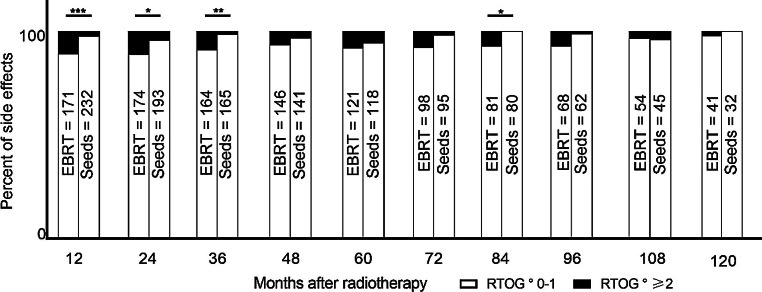
Development of gastrointestinal side effects after treatment with EBRT or seeds over a follow-up period of 120 months. *p < 0.05, **p < 0.01, ***p < 0.001

**Fig. 3:**
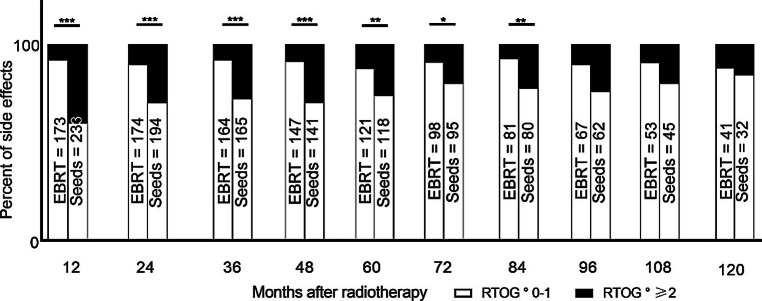
Development of genitourinary side effects after treatment with EBRT or seeds over a follow-up period of 120 months. *p < 0.05, **p < 0.01, ***p < 0.001

The course of side effects over a follow-up period of 120 months is displayed in Figs. 2 and 3. As our goal with this study is to display the differences of EBRT and seeds, we merged both EBRT groups. Side effects are arranged in a group with RTOG grade 0 and 1 and another one with RTOG grade 2 and higher. Thereby, we aim to provide a better overview of the level of occurrence of clinically relevant side effects.

For the first 3 years of follow-up, we observed a significantly higher level of gastrointestinal RTOG grade ≥2 toxicity in patients treated with EBRT. Nevertheless, the highest observed rate of RTOG grade ≥2 toxicity was only 10% of patients treated with EBRT after 24 months of follow-up. From this point on, higher levels of gastrointestinal toxicity declined over time and were almost gone after 120 months of follow-up.

Genitourinary RTOG grade ≥2 toxicity was significantly higher for the first 7 years in patients treated with seeds, up to a maximum of 40% after 12 months of follow-up. While, as for gastrointestinal side effects, also declining over time, 16% of all seeds patients reported RTOG grade ≥2 toxicity after 120 months of follow-up. For EBRT, RTOG grade ≥2 toxicity alternated between 5 and 12% of all patients, without a decline over time. Differences regarding the number of patients at risk between bNED rate and toxicity are due to a lack of documented RTOG grades.

## Discussion

Low-risk prostate cancer can be equally effectively treated in multiple ways, ranging from active surveillance to prostatectomy to radio-oncological treatment with EBRT or brachytherapy [1, 2, 9–11]. Therefore, it is important to help patients find the optimal individual treatment. With our study, we wanted to present an overview of bNED and side effects for EBRT and seeds, based on data representing daily clinical practice.

Concerning bNED in low-risk patients treated with seeds, the reported bNED rates are around 90% after more than 5 years [12–14] and even 95% after 17 years for patients with age below 60 [15]. In our institution, we were able to achieve rates of 94% after 10 years. Given that all the reported brachytherapy treatments were performed by one person, this is possibly due to the reported learning curve of brachytherapy [16–18]. For EBRT, our observed bNED of 94% after 5 years and 87% after 10 years are higher than the results of the CHHiP trial [19] concerning the 74 Gy arm. It is, nevertheless, noteworthy that 44% of our patients treated with 74 Gy received some kind of ADT, while none of the CHHiP trial patients were treated with ADT, thus, possibly, lowering the bNED rate. Compared to a prospective Australian study from 2019 [20] and the MRC RT1 trial [21], our reported bNED is higher, most likely because the former also includes patients treated with 70 Gy, which is known to be insufficient [11, 22], and the latter also includes non-low-risk patients. Regarding the 78 Gy group, our bNED rate of 98% after 5 years is much higher than the reported failure-free rate of 74% by Peeters et al. [23]. It is still noteworthy that Peeters included a large number of high-risk patients. On top of that, failure-free rates were defined using the ASTRO definition and also including clinical failure, as opposed to our bNED definition. Looking at the bNED rates of way above 90% for low-risk prostate cancer achieved by Pasalic et al. [24], with a remarkable follow-up of over 20 years, it is clearly displayed that our 78 Gy group is lacking size, as two events of biochemical failure after 84 and 96 months decrease our bNED rate from over 95% to merely above 80%.

It has to be noted that the high proportion of 87% of patients treated with 3D conformal EBRT no longer matches the reality in our institution, as all prostate cancer patients are nowadays treated with VMAT and hypofractionated radiotherapy according to the CHHiP trial [19] and as displayed by Schörghofer et al. [25] in 20 fractions with 3‑Gy single dose.

Concerning late side effects, we were able to record reduced genitourinary side effects and increased gastrointestinal side effects for EBRT in comparison to seeds. This matches the results of other studies [26–28]. The fact that the side effects diminish over time and approach levels close to the level before treatment is also described by Sanda et al. [27]. Regarding maximum side effects, we observed a significant increase for late genitourinary side effects in patients treated with 78 Gy compared to 74 Gy. This was not the case for late gastrointestinal side effects, most likely due to reduced dorsal PTVs in patients treated with 78 Gy. This PTV reduction seems to have an effect similar to a rectal retractor [29]. VMAT technique, as described by Buschmann et al. [30], is today’s standard of care in our department. Lower toxicity rates for patients treated in our institution with EBRT nowadays can therefore be expected, as IMRT is a predictor for reduced toxicity [26, 31].

The weaknesses of our study are its retrospective nature and the small number of patients treated with 78 Gy. On top of that, 44% of all patients treated with 74 Gy received ADT, which is not recommended as routine therapy for primary low-risk prostate cancer according to today’s standard of treatment [2, 9]. This is due to the fact that ADT was administered by the treating urologist and patients had already started ADT before the first visit to our department of radiation oncology. Leaving the final treatment choice between BT and EBRT to the patient is also a source of possible bias.

On the other hand, our study shows several strengths. It is a monocentric study, which facilitates comparison of side effects, as every side effect is reported in the same way. The large number of patients treated with seeds by only one radiation oncologist also allows a high level of quality in treatment to be assumed, as displayed in a nationwide Japanese study [32]. Moreover, all data collected were the result of daily clinical practice. Therefore, this study does not show any bias through possible study conditions. Beyond that, 17% of all patients included had a follow-up of 10 or more years, allowing sufficient data collection for this study’s statement of bNED. 5 out of 10 biochemical failures after seed implantation were irradiated again, showing that even after failure of the primary treatment, there is still a radio-oncological salvage option, whereas only 1 out of 14 bNED failures after EBRT was once again irradiated.

## Conclusion

Our data show increased gastrointestinal side effects for EBRT and increased genitourinary side effects for seeds. However, the intensity of both types of side effects tends to decrease over time. No significant difference in bNED can be seen between the displayed treatment modalities. Nevertheless, there seems to be a tendency for improved bNED favoring seeds in our data. Therefore, seeds should always be discussed as a valid treatment option for low-risk prostate cancer patients, especially to enable informed decision making for patients in terms of side effects.
